# Discrimination of grasshopper (Orthoptera: Acrididae) diet and niche overlap using next-generation sequencing of gut contents

**DOI:** 10.1002/ece3.1585

**Published:** 2015-07-07

**Authors:** Beverly McClenaghan, Joel F Gibson, Shadi Shokralla, Mehrdad Hajibabaei

**Affiliations:** Department of Integrative Biology, Biodiversity Institute of Ontario, University of GuelphGuelph, N1G 2W1, Ontario, Canada

**Keywords:** Diet analysis, Illumina MiSeq, niche overlap, plants, *rbcLa*

## Abstract

Species of grasshopper have been divided into three diet classifications based on mandible morphology: forbivorous (specialist on forbs), graminivorous (specialist on grasses), and mixed feeding (broad-scale generalists). For example, *Melanoplus bivittatus* and *Dissosteira carolina* are presumed to be broad-scale generalists, *Chortophaga viridifasciata* is a specialist on grasses, and *Melanoplus femurrubrum* is a specialist on forbs. These classifications, however, have not been verified in the wild. Multiple specimens of these four species were collected, and diet analysis was performed using DNA metabarcoding of the gut contents. The *rbcLa* gene region was amplified and sequenced using Illumina MiSeq sequencing. Levins’ measure and the Shannon–Wiener measure of niche breadth were calculated using family-level identifications and Morisita’s measure of niche overlap was calculated using operational taxonomic units (OTUs). Gut contents confirm both *D. carolina* and *M. bivittatus* as generalists and *C. viridifasciata* as a specialist on grasses. For *M. femurrubrum*, a high niche breadth was observed and species of grasses were identified in the gut as well as forbs. Niche overlap values did not follow predicted patterns, however, the low values suggest low competition between these species.

## Introduction

Grasshoppers (Orthoptera) have a wide range of feeding habits, from being strict resource specialists to broad generalists. Understanding the feeding ecology of grasshoppers can provide important insight into the evolution of diet specialization (Otte and Joern 1976; Karpestam and Forsman 2013) and resource partitioning among sympatric species (Krzysik 1979; Behmer and Joern 2008; Masloski et al. 2014). Food choices have been shown to affect fitness traits in grasshoppers, including fecundity, development, and survival (Joern 1979; Ebeling et al. 2013; Harrison et al. 2014).

Subfamilies and species of short-horned grasshoppers (Acrididae) have been previously classified by presumed feeding habit. Joern (1979) designated species of Melanoplinae as specialists on forbs (angiosperms excluding Poaceae, Juncaceae, and Cyperaceae), species of Gomphocerinae as specialists on grasses (Poaceae), and species of Oedipodinae as generalists. Isely (1944) described the mandible morphology of over one hundred species of Acrididae and Tettigoniidae (Orthoptera) and classified them as forbivorous (specialist on forbs), graminivorous (specialist on grasses), or mixed feeding (broad-scale generalists). Four species of Acrididae found in southern Ontario were classified by Isely (1944) as follows: *Melanoplus femurrubrum* (Melanoplinae) is forbivorous, *Chortophaga viridifasciata* (Oedipodinae) is graminivorous, and *Melanoplus bivitattus* (Melanoplinae) and *Dissosteira carolina* (Oedipodinae) are mixed feeders. These species classifications follow the subfamily classifications of Joern (1979) for *M. femurrubrum* and *D. carolina*, but not for *M. bivittatus* and *C. viridifasciata*. More recent studies of diet specialization for these species are also conflicting. Loaiza et al. (2008) describe *M. bivittatus* as a generalist. Tuberville et al. (1996) describe *C. viridifasciata* as a generalist. Previous studies have mainly employed controlled laboratory-based or modified field-based experiments and not direct analysis of wild-caught grasshoppers (e.g., Jonas and Joern 2008).

Conventional methods of grasshopper diet analysis rely on direct observation of feeding behavior, microscopic analysis, and carbon isotope analysis of gut contents (Behmer and Joern 2008; Jonas and Joern 2008; Ibanez et al. 2013; Karpestam and Forsman 2013). DNA sequence analysis allows for the identification of gut contents, including partially digested plants, to the family, genus, or species level (Jurado-Rivera et al. 2009; Navarro et al. 2010; Pompanon et al. 2012; García-Robledo et al. 2013; Kishimoto-Yamada et al. 2013; Heise et al. 2015). Specifically, DNA barcoding uses a standardized region of DNA for species-level identification (Hebert et al. 2003). The DNA barcode region is amplified, sequenced, and identified through comparison to an online database. The introduction of next-generation sequencing technologies, such as the Illumina MiSeq, allows for analysis of bulk samples (e.g., gut contents) containing DNA from multiple individuals, to be characterized at once, broadening the application of these technologies (Shokralla et al. 2012). This DNA metabarcoding approach has shown great potential in the analysis of environmental samples with a wide range of ecological applications including diet analysis (Hajibabaei 2012). Although DNA in food gets degraded as it passes through the digestive tract, partially degraded DNA can still be recovered and identified. For example, Boyer et al. (2013) were able to detect degraded earthworm DNA in the feces of snails and Pegard et al. (2009) were able to detect plant species consumed by livestock from fecal samples. Previous studies have been successful at using DNA metabarcoding for diet analysis in beetles (Kajtoch 2014; Kajtoch and Mazur 2015) as well as grasshoppers (Ibanez et al. 2013).

Plant DNA barcoding typically relies on chloroplast genes, and a two-locus barcode (ribulose-bisphosphate carboxylase gene (*rbcLa*) and maturase K (*matK*)) has been proposed as a DNA barcode for plants (CBOL 2009). While both gene regions have been used previously in diet analysis, it has been noted that most PCR amplification primers for *matK* do not adequately amplify a broad range of plant taxa (Heise et al. 2015). Alternate plant barcode regions, including tRNALeu UAA (*trnL*) and the *trnH-psbA* intergenic spacer, have been used for diet analysis. Both of these markers, however, are hampered by highly variable length and limited public database coverage (Heise et al. 2015), making them particularly poorly suited to use with metabarcoding protocols. The *rbcLa* region is useful for family- and genus-level identification, but does not usually resolve sequences well at the species level (Bafeel et al. 2012; Heise et al. 2015). The analysis of operational taxonomic units (OTUs) can provide higher resolution of the sequence diversity present in the gut even if all OTUs are not identified (Blaxter 2004). This is useful for calculating niche overlap to determine the resource partitioning where species identification is not required.

Here, we use *rbcLa* DNA metabarcoding to determine the diet breadth of four species of grasshoppers in the family Acrididae. We hypothesize that *M. bivittatus* and *D. carolina* are broad-scale generalists while *C. viridifasciata* and *M. femurrubrum* are specialists on grasses and forbs, respectively. We predict that the niche overlap between generalist species is high, the niche overlap is low between specialists, and the niche overlap is intermediate between generalists and specialists. This will allow us to quantitate how resources are partitioned among coexisting grasshopper species.

## Material and Methods

### Field collection

Grasshopper specimens were collected in September 2013 at three locations near Guelph, Ontario, Canada (Little Tract 43° 26.775′ N, 80° 14.861′ W; Starkey Hill 43° 32.712′N, 80° 9.303′ W; University of Guelph Arboretum 43° 32.389′ N, 80° 12.887′W). At Little Tract (LT), four individuals of *Melanoplus bivittata* and three individuals of *Chortophaga viridifasciata* were collected. At Starkey Hill (SH), four individuals of *Dissosteira carolina* and four individuals of *Melanoplus femurrubrum* were collected. At University of Guelph Arboretum (Arb), three individuals of *D. carolina* and two individuals of *C. viridifasciata* were collected. Specimens were preserved in 100% ethanol and stored at −20°C until processing (approximately 4 months).

### Grasshopper identification

A leg was pulled from each individual collected, and DNA was extracted using a Macherey–Nagel nucleospin tissue extraction kit. PCR amplification was performed following Hajibabaei et al. (2012) on the DNA extracts to amplify the cytochrome *c* oxidase subunit I (*COI*) gene region using primers described in Folmer et al. (1994). Amplicons were then subjected to standard Sanger sequencing in an Applied Biosystems 3730XL DNA sequencer. Sequences were identified to species through comparison with the BOLD database (Ratnasingham and Hebert 2007) at a minimum 98% similarity.

### Plant identification

Grasshopper guts (including foregut, midgut, and hindgut) were dissected out of each individual. Samples were homogenized using an MP FastPrep-24, and DNA extractions were performed using a Macherey–Nagel nucleospin tissue extraction kit. PCR amplification was used to amplify the *rbcLa* region (∼550 bp) for plant identification in the gut using the following primers: rbcLa-F ATGTCACCACAAACAGAGACTAAAGC and rbcLa-R GTAAAATCAAGTCCACCRCG (Levin et al. 2003). The PCR solution consisted of 2 *μ*L of DNA template, 17.5 *μ*L of molecular biology grade water, 2.5 *μ*L of 10× reaction buffer (200 mM Tris-HCl, 500 mM KCl, pH 8.4), 1 *μ*L of MgCl_2_ (50 mM), 0.5 *μ*L of dNTPs mix (10 mM), 0.5 *μ*L of the forward primer (10 mM), 0.5 *μ*L of the reverse primer (10 mM) and 0.5 *μ*L of Invitrogen’s Platinum Taq polymerase (5 U/*μ*L). The PCR conditions consisted of 4 min at 94°C, 35 cycles of 30 sec at 94°C, 30 sec at 55°C, and 1 min at 72°C, with a final extension of 10 min at 72°C and held at 4°C. PCR products were visualized on 1.5% agarose gels, and any samples that did have sufficient product were amplified again using the same primers and the same conditions with five extra amplification cycles. All samples showed sufficient PCR products following either the first or second PCR protocol. PCR products were then purified using a Qiagen MinElute PCR purification kit and eluted in 30 *μ*L of molecular biology grade water. Following purification, Illumina sequencing adaptors were added to the *rbcLa* products as described in Wong et al. (2013). This second PCR solution was made following the same protocol as previously described. The PCR conditions consisted of 2 min at 94°C, 35 cycles of 1 min at 94°C, 30 sec at 48°C, and 1 min at 72°C, with a final extension of 5 min at 72°C and held at 4°C. Products were visualized on a 1.5% agarose gel. As before, if any samples did not amplify well, they were amplified again following the PCR protocol with five extra cycles The amplicons for each individual were then purified, quantified, and sequenced using the Illumina MiSeq.

The Illumina sequences were filtered for quality and trimmed using PRINSEQ v0.20.4 (Schmieder and Edwards 2011) with a minimum 20 phred quality score, a window of 10, and a step of 5. Due to the read length limitation, the *rcbLa* region was analyzed as two fragments whose ends were paired to create a 550-bp sequence and clustered at 98% similarity. Sequences were identified using the MEGABLAST algorithm (Zhang et al. 2000) against a reference library of all *rbcLa* sequences downloaded from the GenBank database (March 17, 2014) with a minimum *E*-value of 1e-20. Sequence matches for each cluster were summarized using MEGAN 5 (Huson et al. 2011). Previous studies (e.g., Bokulich et al. 2013) have shown that with Illumina MiSeq sequencing, it is advisable to discard extremely rare sequences from metabarcoding analyses. For this reason, any cluster with less than ten sequences within an individual was omitted from subsequent analysis. The results from this analysis were used for niche breadth calculations.

All Illumina sequences from all twenty specimens were also compiled and clustered into 97% similarity OTUs using UCLUST software (Edgar 2010). All clusters including at least 100 sequences were included in a sequence number per specimen by OTU matrix. This matrix was used to calculate niche overlap and also subjected to a nonmetric multidimensional scaling (nMDS) analysis using the *vegan* package in R (Oksanen et al. 2013). This analysis uses a square root transformation of the matrix to generate Bray–Curtis (*i.e*., rank-based abundance) dissimilarities between specimens. These dissimilarities are then plotted in two dimensions.

### Data analysis

Species niche breadth was calculated using Levins’ measure (1968):

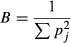


*B* = Levins’ measure of niche breadth

*p*_*j*_ = fraction of items in the diet that are resource *j*

and the Shannon–Wiener measure (Shannon 1948):

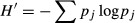


*H*′ = Shannon–Wiener measure of niche breadth

*p*_*j*_ = proportion of individuals using resource *j*

Levins’ measure emphasizes the most frequently used resources while the Shannon–Wiener measure emphasizes rarities in the diet (Krebs 1999). Both indices were used to compare niche breadth values with emphasis on rare and frequently used plants and to determine whether these different measures led to major differences in grasshopper species niche breadth. A t-test was used to compare Shannon–Wiener measures of niche breadth between species following Brower et al. (1997).



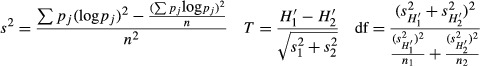


*p*_*j*_ = proportion of individuals using resource *j*


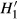
, 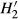 = Shannon–Wiener measure of niche breadth of species 1 and 2


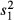
, 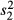 = variance of 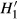 and 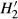


*n *= total number of resources used by all individuals

*T* = *T* statistic

df = degrees of freedom

These calculations were performed on sequences identified to the family level and the highest level of resolution where the majority of sequences were identified.

Species niche overlap was calculated using Morisita’s measure (1959):

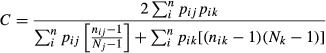


*C* = Morisita’s measure of niche overlap between species *j* and *k*

*p*_*ij*_ = proportion resource *i* is of the total resources used by species *j*

*p*_*ik*_ = proportion resource *i* is of the total resources used by species *k*

*n*_*ij*_ = number of individuals of species *j* that use resource category *i*

*n*_*ik*_ = number of individuals of species *j* that use resource category *k*

*N*_*j*_, *N*_*k*_ = total number of each species in sample

Morisita’s measure has been suggested as the least biased measure of niche overlap (Krebs 1999). Niche overlap measures were calculated using unidentified OTUs.

Sequences that were identified to species were cross-referenced with the Plants of Canada Database (Canadian Food Inspection Agency 2013), and any plant species that was not found in the database was not reported here.

## Results

The results of sequencing, filtering, clustering, and taxonomic identification are displayed in Table1. The majority of sequences were identified to family level; however, genus-level identification was variable, and species-level identification was achieved in <5% of the filtered sequences for most samples. Poaceae was detected in all species. Poaceae was the only major family identified from *C. viridifasciata* gut sequences. *M. bivittatus*, *D. carolina* and *M. femurrubrum* all had between three and seven major plant families present in the gut. Polygonaceae was the only family other than Poaceae that was found in all three of these species.

**Table 1:** Summary of Illumina *rbcLa* sequence output, including quality filtering, 98% similarity clustering, and percentage identified to family level, genus level, and species level

Individuals	Species	Total # of Seqs	# of Seqs filtered	# Clusters	% Seqs ID’d to family	% Seqs ID’d to genus	% Seqs ID’d to species
Arb 2I	*C.v*.	117,233	47,419	18,183	97.91	4.94	1.67
Arb 2J	*C.v*.	67,050	32,029	8388	98.57	2.02	1.38
Arb A	*D.c*.	118,210	65,683	22,703	99.59	84.19	1.45
Arb B	*D.c*.	282,533	38,330	11,621	89.99	5.18	4.96
Arb C	*D.c*.	373,372	12,791	7494	63.43	51.88	1.00
LT A	*C.v*.	340,301	26,910	10,731	86.45	17.09	1.46
LT B	*C.v*.	302,982	30,459	9715	93.22	2.18	1.43
LT C	*C.v*.	101,816	50,890	14,378	96.10	3.22	1.10
LT D	*M.b*.	89,875	36,898	15,256	94.45	8.50	3.64
LT F	*M.b*.	102,254	65,156	21,351	98.95	87.22	0.68
LT G	*M.b*.	105,870	64,438	20,625	94.48	0.70	0.35
LT H	*M.b*.	253,502	38,223	13,000	93.70	9.63	2.03
SH A	*M.f*.	140,212	99,031	38,627	95.82	74.51	2.40
SH B	*M.f*.	593,645	31,878	12,803	81.23	2.98	2.05
SH C	*M.f*.	96,266	27,110	10,982	71.39	8.40	6.99
SH D	*M.f*.	118,638	51,475	14,609	96.17	1.94	1.43
SH E	*D.c*.	64,666	44,582	16,207	99.82	95.47	0.13
SH F	*D.c*.	305,822	37,731	16,186	91.16	71.41	1.82
SH G	*D.c*.	395,608	64,258	21,717	96.27	2.27	1.72
SH H	*D.c*.	162,668	45,754	22,269	88.64	76.62	36.50

*M.f., Melanoplus femurrubrum; M.b., Melanoplus bivittata; D.c., Dissosteira Carolina; C.v., Chortophaga viridifasciata*.

**Figure 1:**
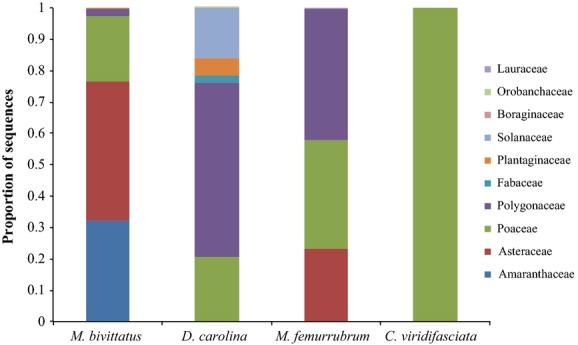
Proportion of quality trimmed and filtered *rbcLa* sequences from the gut contents of four species of grasshoppers identified to each plant family in all individuals combined.

**Figure 2:**
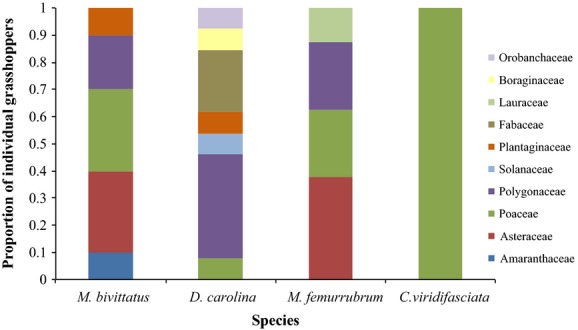
Proportion of individual grasshoppers in which each plant family was identified in the gut for four grasshopper species.

Family-level sequence identifications are displayed as a proportion of sequences identified to each plant family for each species of grasshopper in Figure1. These proportions were used to calculate Levins’ niche breadth. Figure2 displays family-level sequence identification as a proportion of individuals of each grasshopper species with that plant family identified in the gut. These proportions were used to calculate the Shannon–Wiener measure of niche breadth. Both Levins’ and Shannon–Wiener niche breadth measures are displayed in Table2 for each species. With both measures, *C. viridifasciata* had the lowest niche breadth. Results from t-tests performed on the Shannon–Wiener measure between all species are shown in Table3. There was a significant difference between the niche breadth of *C. viridifasciata* and all three other grasshopper species. There was also a significant difference between the niche breadth of *M. femurrubrum* and *D. carolina*. Morisita’s measure of niche overlap between all species at the OTU level is shown in Table4. The highest niche overlap was seen between *M. femurrubrum* and *D. carolina,* while the lowest niche overlap was seen between *M. bivittatus* and *D. carolina. Melanoplus femurrubrum* had consistently high niche overlap with all three other species.

**Table 2:** Levins’ and Shannon–Wiener (S-W) measures of niche breadth for four grasshopper species

	Levins’	S-W
*M. bivittatus*	2.903	0.639
*M. femurrubrum*	2.854	0.545
*C. viridifasciata*	1	0
*D. carolina*	2.639	0.866

**Table 3:** Results of t-test between Shannon–Wiener niche breadths at the family level in four grasshopper species

	*M. femurrubrum*	*C. viridifasciata*	*D. carolina*
*M. bivittatus*	*P* > 0.05	***P***** < 0.05**	*P* > 0.05
	*t* = 0.79	***t*** = 7.79	*t* = 1.77
	df = 4	df = 5	df = 11
*M. femurrubrum*		***P***** < 0.05**	***P***** < 0.05**
		***t*** = 6.33	***t*** = 2.45
		df = 4	df = 4
*C. viridifasciata*			***P***** < 0.05**
			***t*** = 8.78
			df = 7

Significant differences are in bold.

**Table 4:** Morisita’s measure of niche overlap between four grasshopper species at the operational taxonomic unit (OTU) level

	*M. femurrubrum*	*C. viridifasciata*	*D. carolina*
*M. bivittatus*	0.0427	0.0194	0.0108
*M. femurrubrum*		0.0721	0.172
*C. viridifasciata*			0.0261

**Figure 3:**
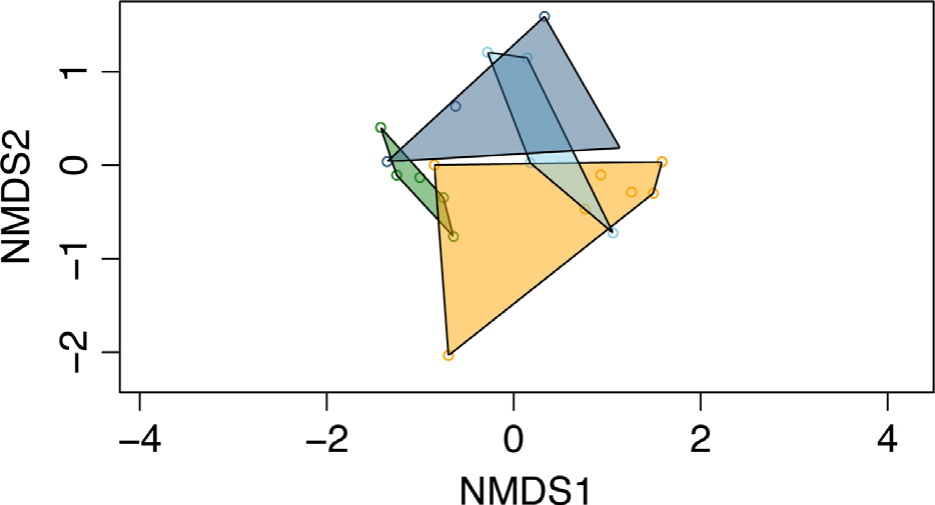
Nonmetric multidimensional scaling (nMDS) analysis of OTUs generated from all *rbcL* sequences recovered from the guts of all twenty grasshopper specimens. Polygons include all points corresponding to all specimens of each species. Green – *C. viridifasciata*; orange – *D. carolina*; light blue – *M. bivittatus*; dark blue – *M. femurrubrum*.

The nMDS plot of OTUs containing at least 100 sequences (*n* = 74) is shown in Figure3 (stress value −0.13). This nonmetric plot of community composition based on all recovered sequences recovers minimal overlap between species. There is no overlap at all between *M. femurrubrum* and *D. carolina*. There is some overlap of *M. bivittatus* with both *M. femurrubrum* and *D. carolina*. There is also some small overlap of *C. viridifasciata* with both *M. femurrubrum* and *D. carolina*.

Measures of niche breadth and overlap consider all individuals in a consumer species together. Not all individuals of a grasshopper species had the same resource plant families or OTUs identified in the gut. *C. viridifasciata* was the only species in which all individuals had the same resource plant families in the gut since only a single family, Poaceae, was present. The other three grasshopper species had between four and seven plant families identified in the gut; however, each individual of these species had only one or two families identified in the gut. The majority of plant families were found in more than one individual of each grasshopper species. At the OTU level, there were also considerable differences between individuals (Table5). Most OTUs were found in only one or two individuals of each species. All individuals had between 3 and 37 OTUs present with an average 19.25 OTUs present in each individual. This suggests that the individual niche breadth is much smaller than the species niche breadth for each grasshopper species. Species-level identification represented only a small fraction of the total sequences (Table2). Plant species identified in the gut of each grasshopper species are found in the online supporting material. One species of introduced ornamental grass, *Arrhenatherum elatius*, was found in the gut of all four grasshopper species.

**Table 5:**
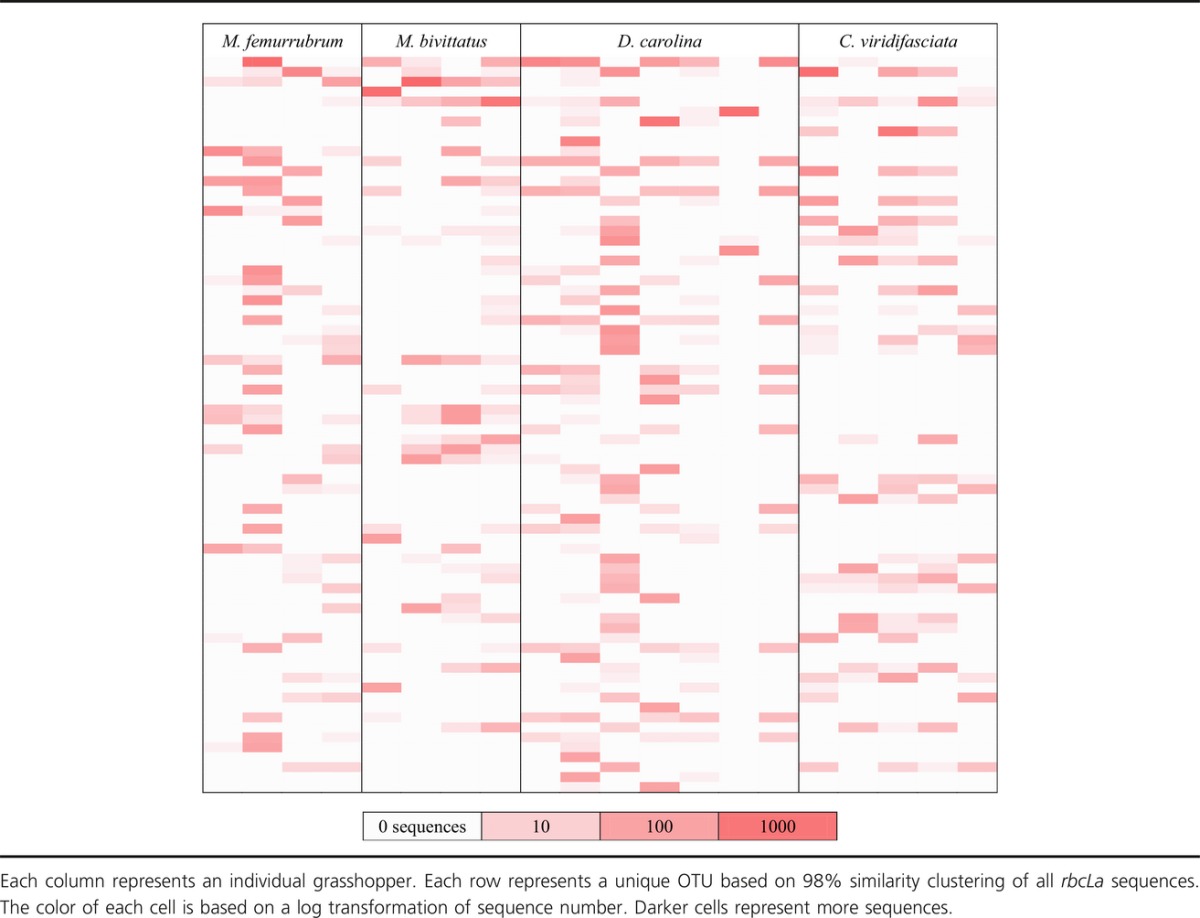
Heat map of *rbcLa* OTU recovery for the gut contents of four species of grasshopper

## Discussion

Molecular analysis of gut contents provides a clear picture of the feeding habits of the four species studied. *Dissosteira carolina*, described as a generalist by both Joern (1979) and Isely (1944), had high niche breadth values and multiple plant families present in the gut, making it a generalist. *Melanoplus bivittatus*, described as a forb specialist by Joern (1979) and a generalist by Isely (1944) and Loaiza et al. (2008), also had high niche breadth values and multiple plant families present in the gut, making it a generalist. *Chortophaga viridifasciata*, described as a generalist by Joern (1979) and Tuberville et al. (1996) and a grass specialist by Isely (1944), had the smallest niche breadth, and Poaceae was the only family identified in the gut. *Melanoplus femurrubrum*, described as a forb specialist by Joern (1979) and Isely (1944), has a relatively high niche breadth (Shannon-Wiener: 0.545) and was much more similar to the two generalist species (S-W: 0.639 and 0.866) than to the specialist (S-W: 0). Furthermore, both forbs and grasses were identified from the gut contents of *M. femurrubrum* indicating that it does not feed exclusively on forbs. Our niche breadth predictions were true for three species: *C. viridifasciata* had a small niche breadth, and *D. carolina* and *M. bivittatus* had high niche breadths. *M. femurrubrum* had a higher niche breadth than predicted. Niche breadth values were similar to those found in diet analyses of other Orthoptera species using morphological identification of fecal contents (Capello et al. 2012).

A previous investigation of six species of Orthoptera diets (Capello et al. 2012) recovered niche overlap values much higher (range: 0.015–0.842) than those recovered here (range: 0.011–0.172). While these previous measures were calculated with Pianka’s (1974) overlap measure, this has been demonstrated to be comparable to the Morisita measure employed here (Goodyear 1992; Qi et al. 2009). Our lower values are likely due to the greater number of diet components included in analysis. Whereas previous experiments utilized only fourteen morphologically identifiable plant species, our analysis employed 74 genetically distinct OTUs. The lowest niche overlap was observed between the generalists *M. bivittatus* and *D. carolina*, suggesting reduced interspecific competition for resources. The highest niche overlaps were between *M. femurrubrum* and each of the other three species. The relatively low overlap values suggest low competition between species for local resources. The nMDS plot (Fig.3) is useful in demonstrating the low niche overlap we observed. The distinct areas occupied by the four species on the graph represent the distinct sets of plant resources used by these species. We also observed patterns of niche overlap that did not follow our predictions; however, the results suggest that among coexisting grasshoppers, there is resource partitioning to reduce competition.

Our estimates of niche breadth give important insight into the local feeding ecology and use of resources by these grasshoppers. Due to the small number of individuals processed here, they must be assumed, however, to be underestimates of the niche breadth of the species as a whole. Diet breadth may change with season as is seen in other generalist herbivores (Stolter et al. 2013). The diet may also be different across the range of these species as plant species availability and environmental conditions differ (Kajtoch 2014; Kajtoch and Mazur 2015). Changes in diet across a species’ range have been observed in other grasshopper species (Franzke et al. 2010). Additionally, DNA analysis of diet cannot reflect diet over a large temporal scale as it is limited by the transit time of food through the digestive tract of grasshoppers – estimated at 6 h (Chapco and Kelln 1994).

The *rbcLa* region is effective for family-level identification; however, it performs poorly at lower taxonomic levels, as observed in this study. Other studies have shown similar patterns of plant identification using *rbcLa* (CBOL 2009; Burgess et al. 2011; Wallace et al. 2012). Accurate taxonomic assignment using DNA barcodes is also linked to availability of reference sequences from identified species. While the reference library available for *rbcLa* is larger than many other plant markers, higher resolution in identifying gut contents could be accomplished by improving the reference library or using additional markers, such as *matK* (megakaryote-associated tyrosine kinase gene in the chloroplast) or ITS (ribosomal internal transcribed spacer) (Pompanon et al. 2012). The *rbcLa* gene region has previously been used in diet analysis studies of insects with successful family-level identifications (Kishimoto-Yamada et al. 2013). As a relatively long gene region (∼550 bp), *rbcLa* may not amplify well for degraded sample tissues. However, we have demonstrated the ability to use Illumina MiSeq sequencing to recover the full *rbcLa* region from gut contents of grasshoppers.

The number of sequences assigned to each taxon may not accurately reflect relative abundance of a plant in the gut due to biases during PCR amplification, quality filtering biases, and minimum read length (Deagle et al. 2013). In this study, we had very high depth of coverage and individuals had between one and three OTUs present at *very* high sequence numbers that would not have been generated purely by bias. However, calculating two values of niche breadth, one which used the number of sequences for each family and one which did not use the number of sequences, helped to account for any biases in the number of sequences. The Shannon–Wiener measure used the proportion of individuals the family was identified in and yielded similar, although slightly different, results to Levins’ measure which used the number of sequences.

In this study, we were able to draw preliminary conclusions regarding the feeding ecology of four grasshopper species. We provide evidence that three of these grasshopper species are feeding according to the generalist and specialist groups to which they have previously been classified. The fourth species, *M. femurrubrum*, previously classified as a specialist on forbs, would be better classified as a generalist. Furthermore, we were able to show that these three sympatric generalist grasshoppers are able to reduce competition by making use of different resources. We also provide some insight into the use of the full-length *rbcLa* region for future diet analysis.

## Data accessibility

DNA sequences generated in this study have been deposited to DRYAD and are accessible under accession number – doi:10.5061/dryad.r8c3b.

## Supporting Information

**Table S1**. Plant species identified in the gut of each grasshopper species with number of sequences.
