# Supplementary material for: Discrimination of grasshopper (Orthoptera: Acrididae) diet and niche overlap using next-generation sequencing of gut contents
Source: Ecol Evol. 2015 Jul 7;5(15):3046–55. doi: 10.1002/ece3.1585 (PMC4559048; doi:10.1002/ece3.1585)
Supplement: Supplementary file 1 [file ece30005-3046-sd1.docx]

**Supporting Material: Table S1.** Plant species identified in the gut of each grasshopper species with number of sequences. Plants that are classified as grasses (Poaceae) are marked in green, plants classified as forbs are marked in red and plants that are neither grasses nor forbs are in white.

|  | **C. viridifasciata** | **M.bivittatus** | **D. carolina** | **M. femurrubrum** |
| --- | --- | --- | --- | --- |
| **Agrostis stolonifera** |  |  |  | 8 |
| **Agrostis scabra** | 5 |  | 7 |  |
| **Apera interrupta** | 237 |  | 13 | 223 |
| **Arctagrostis latifolia** |  |  | 59 |  |
| **Arrhenatherum elatius** | 215 | 78 | 85 | 177 |
| **Briza maxima** | 5 |  | 269 |  |
| **Bromus erectus** | 18 | 761 | 4 |  |
| **Bromus porteri** | 31 |  | 12 |  |
| **Deschampsia cespitosa** |  |  |  | 15 |
| **Elymus sibiricus** | 143 | 108 | 28 |  |
| **Festuca brachyphylla** | 17 |  |  |  |
| **Lagurus ovatus** |  |  | 9 | 15 |
| **Leymus arenarius** | 44 | 6 |  |  |
| **Milium effusum** | 237 |  | 19 | 225 |
| **Oryzopsis asperifolia** | 30 |  | 18 |  |
| **Phalaris arundinacea** | 45 |  |  |  |
| **Phleum pratense** | 5 |  | 141 | 40 |
| **Poa annua** | 9 |  |  | 5 |
| **Poa pratensis** | 323 |  | 231 | 174 |
| **Populus tremuloides** | 7 |  |  |  |
| **Agalinis maritima** |  |  | 6 |  |
| **Atriplex powellii** |  | 15 |  |  |
| **Axyris amaranthoides** |  | 5 |  |  |
| **Datura stramonium** |  |  | 1148 |  |
| **Fallopia dumetorum** |  |  | 11 |  |
| **Lapsana communis** |  |  |  | 5 |
| **Linaria vulgaris** |  | 5 |  |  |
| **Mycelis muralis** |  |  |  | 63 |
| **Oxyria digyna** |  |  | 5 |  |
| **Pedicularis lanata** |  |  | 6 |  |
| **Plantago rugelii** |  |  | 33 |  |
| **Solanum nigrum** |  |  | 8 |  |
| **Trifolium pratense** |  |  | 316 |  |
| **Trifolium repens** |  |  | 85 |  |
